# Dr. Byron Clifford Cheesman

**Published:** 1913-06

**Authors:** 


Dr. Byron Clifford Cheesman, P. & S. Baltimore, 1880, died at Watertown, N. Y., April 3, aged 56.

